# Critical care resources in the Solomon Islands: a cross-sectional survey

**DOI:** 10.1186/1472-698X-12-1

**Published:** 2012-03-01

**Authors:** Mia Westcott, Alexandra LC Martiniuk, Robert A Fowler, Neill KJ Adhikari, Tenneth Dalipanda

**Affiliations:** 1 Bachelor of Medicine Candidate (2012), University of Newcastle, University Drive, Callaghan, NSW 2308, Australia; 2 George Institute for Global Health, PO Box M201, Missenden Road Sydney, NSW 2050, Australia; 3 Faculty of Medicine, University of Sydney, Sydney, NSW 2006, Australia; 4 Dalla Lana School of Public Health, University of Toronto, 155 College St, Toronto, ON M5T 3M7, Canada; 5 Trauma, Emergency and Critical Care Programme, Sunnybrook Health Sciences Centre, Room D1.08 2075 Bayview Ave, Toronto, ON M4N 3M5, Canada; 6 Interdepartmental Division of Critical Care, University of Toronto, 155 College St, Toronto, ON M5T 3M7, Canada; 7 Department of Critical Care Medicine, Sunnybrook Health Sciences Centre, Room D1.08 2075 Bayview Ave, Toronto, ON M4N 3M5, Canada; 8 Director of Public Health and Primary Health Care, Solomon Islands Ministry of Health and Medical Services, PO Box 349 Honiara, Solomon Islands, USA

**Keywords:** Critical care, Critical illness, Solomon Islands, Lower and middle income countries

## Abstract

**Background:**

There are minimal data available on critical care case-mix, care processes and outcomes in lower and middle income countries (LMICs). The objectives of this paper were to gather data in the Solomon Islands in order to gain a better understanding of common presentations of critical illness, available hospital resources, and what resources would be helpful in improving the care of these patients in the future.

**Methods:**

This study used a mixed methods approach, including a cross sectional survey of respondents' opinions regarding critical care needs, ethnographic information and qualitative data.

**Results:**

The four most common conditions leading to critical illness in the Solomon Islands are malaria, diseases of the respiratory system including pneumonia and influenza, diabetes mellitus and tuberculosis. Complications of surgery and trauma less frequently result in critical illness. Respondents emphasised the need for basic critical care resources in LMICs, including equipment such as oximeters and oxygen concentrators; greater access to medications and blood products; laboratory services; staff education; and the need for at least one national critical care facility.

**Conclusions:**

A large degree of critical illness in LMICs is likely due to inadequate resources for primary prevention and healthcare; however, for patients who fall through the net of prevention, there may be simple therapies and context-appropriate resources to mitigate the high burden of morbidity and mortality. Emphasis should be on the development and acquisition of simple and inexpensive tools rather than complicated equipment, to prevent critical care from unduly diverting resources away from other important parts of the health system.

## Background

Despite a large volume of critical care research in high-income countries over the last two decades--on epidemiology and therapies for common syndromes such as sepsis and acute lung injury, organisation of intensive care units (ICUs) and models of care delivery, and knowledge translation--virtually no research has occurred in settings with the greatest burden of illness and least capacity for care [1,2].

In developed countries, caring for critically ill patients involves a coordinated system of triage, emergency management and ICUs [3]. Such care is viewed as complex and unaffordable for many LMICs, yet much of this burden of illness occurs among children and young adults with reversible illness and a good potential for recovery [3,4]. Acute care is being increasingly recognised as complementary [1], and indeed necessary, to meet the millennium development goals of reducing acute illness, morbidity, and mortality in women and children.

Located in the south Pacific about 1,800 kilometres north-east of Australia, the Solomon Islands has a population of 515,870 [5], spread over 9 provinces and 350 populated islands [6,7]. The Solomon Islands is a "least developed country" (LDC) [8], defined by low income, human resource weakness and economic vulnerability, and is ranked 123 of 169 countries on the United Nations Development Program (UNDP) Human Development Index [9]. Life expectancy at birth is 69 years for males and 72 for females [10]. In 2009, health expenditure represented 5.4% of the gross domestic product (GDP), or $147 international dollars per person [10].

There is one publicly funded, tertiary care hospital in the Solomon Islands, the National Referral Hospital, in the capital of Honiara. Seven of the nine provinces also have public hospitals, and there are four privately funded hospitals [5,11]. According to Ministry of Health and Medical Services data, in 2011 there were a total of 130 doctors, 913 nurses and 146 midwives employed by the government [5], with a ratio of 19 doctors per 100,000 population and 145 nurses and midwives per 100,000 population [12].

In high-income countries, care of the critically ill comprises a large proportion of healthcare spending, yet low-income countries such as the Solomon Islands may have a greater burden of critical illness and little infrastructure to provide care [13]. The objectives of this needs assessment project were to learn what leads to critical illness, resources available and resources needed in LMICs such as the Solomon Islands, an essential first step toward improving care for the sickest of patients.

## Methods

This study used a mixed methods approach, including a cross sectional survey of respondents' opinions regarding critical care needs, ethnographic information and qualitative data. Participants included health care providers from three hospitals across the Solomon Islands. Participants were selected to complete the survey based on factors including: level of exposure to critically ill patients (for example doctors who regularly care for critically ill patients such as general physicians, surgeons and accident and emergency doctors); the appropriate level of knowledge to answer questions regarding medications, equipment and common presenting illnesses; and English skills.

Qualitative data was drawn from survey questions with open-ended, qualitative responses.

Information was also obtained from previously published literature to further inform findings. MEDLINE was searched using the following terms: Solomon Islands, Pacific countries, developing countries, acute care, critical care, and critical illness. Additional literature was found from the reference lists of retrieved publications. We also examined the World Health Organisation (WHO) web site [5,10,12] and the Solomon Islands National Health Strategic Plan [14]. Further discussion is complemented by clinical experience and experience working in hospitals in the Solomon Islands.

### Procedures and study setting

#### Survey development

A survey to gather information on barriers and facilitators to providing critical care in LMICs was developed by two authors (NA, RF) using purposive sampling of health care practitioners (e.g. physicians, nurses, nursing and physician assistants, and governmental health personnel) who interact with acutely ill patients. The survey defined a "critically ill patient" as requiring very frequent monitoring or active treatment of failing organs, without which the patient would likely die. Such a patient may receive such care outside of a hospital, during transportation to a hospital, or in a specialised part of the hospital. We followed rigorous survey development methods, including questionnaire *development *(item generation, item reduction, formatting, pre-testing) and *testing *(clinical sensibility, reliability and validity); before questionnaire administration, according to previously published guidelines [15]. To aid with development, an advisory panel of 15 experts in critical care medicine and survey methodology from developed and developing countries generated, reduced and formatted items of interest along question stems with Likert response frames. Likert responses were anchored by 'very uncommon', 'very uncommonly used', 'very difficult', 'very little help' (score 1) and 'very common', 'very commonly used', 'very easy', and 'major help' (score 5). Next we performed pilot testing (of flow, salience, acceptability and ease of completion) and formal clinical sensibility (comprehensiveness, clarity and face validity) testing prior to final survey formatting. The survey was pilot tested specifically in the Solomon Islands prior to its use in this study. The survey collected information on respondent demographics, usual presentation of critically ill patients to medical attention, most common conditions leading to critical illness, accessibility of critical care resources, the perception of benefit for increased access to specific resources, in addition to free text answers to specific questions.

#### Administration

Surveys were administered over a two-month period (October and November) in 2010 by one of the authors (MW) to 21 health care providers at three hospitals. Respondents were given a brief explanation of the objectives of the survey and basic instructions on how to fill it out, and completed the survey in their own time. These were then collected in person by MW. Respondent role (e.g. consultant, registrar) was not provided in this paper to ensure confidentiality of responses. Several respondents answered questions with Not Applicable (N/A), which we interpreted to mean 'Not available' or 'Does not apply' depending on the question. We assigned such responses a Likert value of 0. Entirely blank item responses were recorded but were deducted from the total number of responses (N) in order to calculate the quoted averages and those shown in tables and graphs.

#### Analysis

For this pilot needs assessment study, a formal sample size calculation was not undertaken. We anticipated 15-20 respondents spread across one urban and two rural locations in the Solomon Islands. Descriptive analyses of survey responses were conducted and frequencies are reported. Survey questions with open-ended, qualitative responses were reviewed for key themes and quotations to illustrate those key themes are presented. All quantitative analyses were performed using Microsoft Office Excel 2007 (Microsoft, Redmond, USA).

#### Ethics

Human Research Ethics Approval for research enrolling clinicians and policy makers was obtained from the University of Sydney Ethics Board. Informed consent was obtained from respondents at the time of survey administration. Respondents desiring knowledge of the survey's findings were given the opportunity to receive the results upon the project's completion.

## Results

### Location, profession and distribution of respondents

We administered the survey in three hospitals (Additional file 1): a tertiary referral hospital (National Referral Hospital, 280 beds, 40-50 doctors, 300 nurses, no ICU) in the capital city; a district hospital, (Helena Goldie Hospital, 78 beds, 4 doctors, 35 nurses, no ICU but two 'acute' beds); and another district hospital (Gizo Hospital, 60 beds, 4 doctors, 30-50 nurses, no ICU). Surgery is performed at all three hospitals surveyed, but Helena Goldie Hospital and Gizo Hospital only provide basic surgical procedures, with many surgical cases being referred to National Referral Hospital.

Twenty-one surveys were distributed, of which 20 were returned completed. One was not completed due to the physician's work commitments. At the National Referral Hospital, surveys were completed by 9 physicians (20% of total physicians at the hospital) and 1 nurse. At each of Helena Goldie Hospital and Gizo Hospital, 4 physicians (100% of total physicians at these hospitals) and 1 nurse completed surveys. In all, 17 (85%) of respondents were physicians (8% of the total number of physicians in the Solomon Islands), and 12 (60%) of respondents were male. Respondents had a median of 7.5 years (range 1-32) of health care experience. Experience in caring for critically ill patients was a median of 4.5 years (range 1-18). Among the 20 surveys completed, approximately 8% of the questions had missing data.

### Patient population, resources available and treatments offered

**Figure 1:**
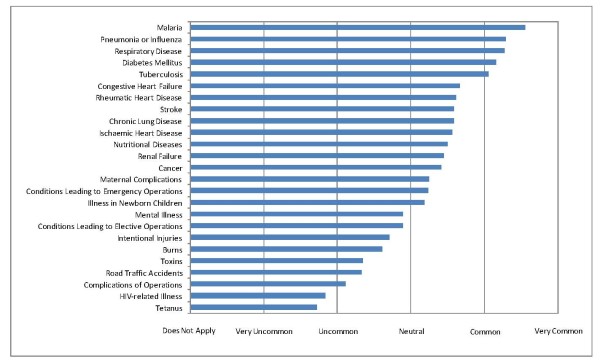
**Conditions leading to the treatment of critical illness**.

Approximately 0-20% of all patients seen (0-5 patients per month) at participating centres were perceived to be critically ill (Table 1). Most patients were adults, but among children, 1-12 months was the most common age (Table 1). The most common conditions leading to critical illness are malaria, diseases of the respiratory system including pneumonia and influenza, diabetes mellitus, and tuberculosis (Figure 1). Complications of surgery and trauma less frequently result in critical illness (Figure 1).

**Table 1:** Proportion and age of patients with critical illness in the Solomon Islands

Proportion of patients treated in last 1 year with critical illness	N (N/19*%)
0-20%	12 (63%)

21-40%	5 (26%)

41-60%	1 (5%)

61-80%	0 (0%)

81-100%	1 (5%)

**Number of patients treated who had critical illness in last 1 month**	N (N/15*%)

0 to 5	11 (73%)

6 to 10	2 (13%)

> 10	2 (13%)

**Average age of critically ill patients in last 1 month (adults)**	N (N/14*%)

17-20 years	2 (14%)

21-30 years	5 (36%)

31-40 years	4 (29%)

41-60 years	3 (21%)

61-80 years	0 (0%)

> 80 years	0 (0%)

**Average age of critically ill patients in last 1 month (children)**	N (N/14*%)

Neonate	3 (21%)

Infant	8 (57%)

Child	0 (0%)

Adolescent	1 (7%)

Not applicable	2 (14%)

* Variation in denominators is due to missing data by survey item

**Figure 2:**
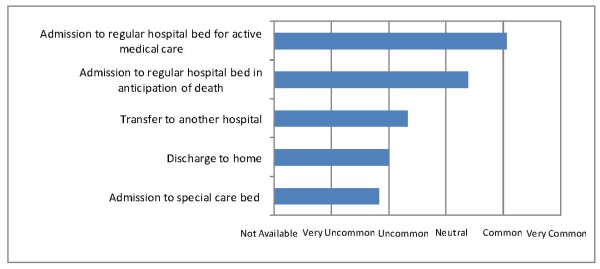
**Likelihood of treatment plan for a critically ill patient**.

**Figure 3:**
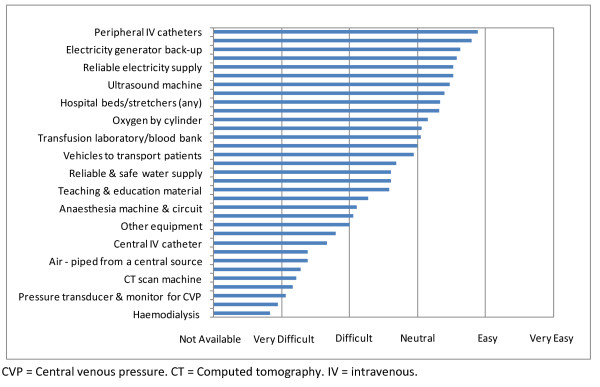
**Ease of accessibility to equipment**.

The most common treatment plan for critically ill patients was to admit to a regular hospital bed for active medical care (Figure 2). There were two 'acute care beds' at Helena Goldie Hospital, but there was no designated place to treat critically ill patients at National Referral Hospital or Gizo Hospital, thus admission to a special care bed was uncommon. There was broad accessibility to basic medical equipment, such as peripheral intravenous catheters and electricity generator back-up, but others such as a blood bank and patient transport vehicles were in limited access, and many not available at all (e.g. invasive pressure monitoring, organ support with haemodialysis, and diagnostic imaging with computed tomography scan) (Figure 3).

**Figure 4:**
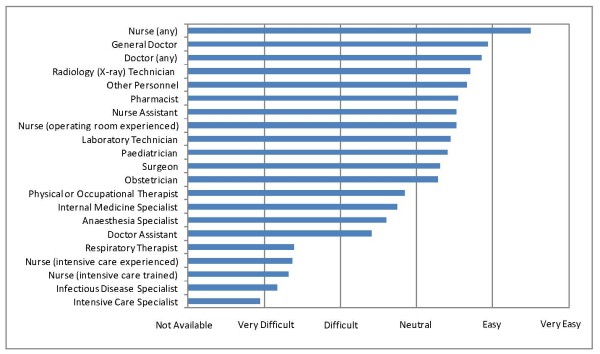
**Ease of accessibility of personnel**.

**Figure 5:**
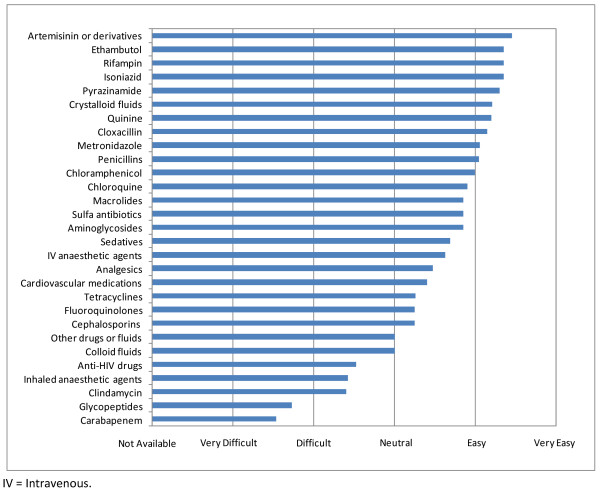
**Ease of accessibility of medications**.

**Figure 6:**
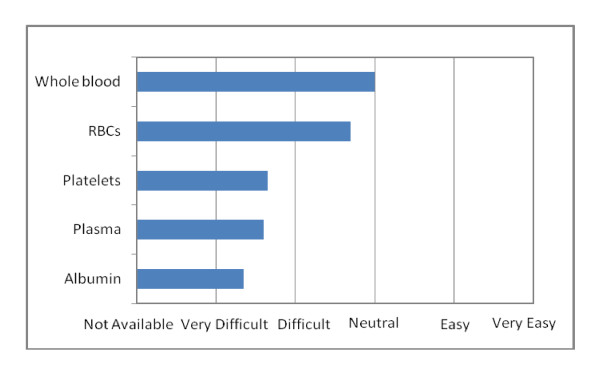
**Ease of accessibility of blood products**.

Nurses were the most accessible of health care personnel but there was limited availability of critical care nurses or physician specialists (Figure 4). The anti-malarial drug (artemisin) and anti-tuberculosis drugs (ethambutol, rifampin, isoniazid and pyrazinimide) were easily accessible, but access to broad-spectrum antibiotics was limited (Figure 5). Blood products were difficult to access, with whole blood perceived to be the most accessible (Figure 6). Access to health information including reliable internet access was difficult, with access to medical journals perceived to be the most difficult.

Broader access to all resources was perceived to have the potential to improve the care and outcome of critically ill patients. Resources perceived to be of greatest potential included broader access to equipment such as oxygen saturation monitors; increased access to human resources such as intensive care-trained staff, including doctors, nurses and anaesthetists; intensive care space physical facilities; medications and blood products; and specific health information.

### Qualitative responses

#### Access to critical care

Decisions to stop or limit critical care were based on multiple factors, including the availability of resources, the family's wishes and doctor's opinion.

One respondent described the decision-making process as follows:

"Decisions are made in a stepwise/referral manner. Upon arrival at the emergency department the doctors provide available acute care before referral to possible advanced care [from a] specialist team since there is no ICU. Consultants make further decision if overseas care is required. Families often decide to stop/limit care. The longer critically ill patients are kept, the more likely to stop [care]. Politics play a major role in [availability] of advanced care."

Other respondents outlined the availability of resources and inability to finance care as influencing the decision to stop or limit care, including the health facility lacking appropriately trained personnel, equipment and treatment options.

Prioritisation of distribution of resources was identified as an important factor in access to critical care, with access limited by scant health resources in the Solomon Islands:

"Multiple factors contribute but the main factor at the moment is [the] financial implications of sustaining an ICU. A single patient in ICU can take up more time and resources that could have been allocated to a greater number of patients who do not need ICU treatment and would in the long run have a better prognosis. At the moment we just could not have both."

#### Suggestions for improved resources

Respondents provided comments and suggestions on improving the care of critically ill patients in their hospital. Further training in the area of intensive care was a key factor identified in order to improve the care of critically ill patients, with comments including:

"Train some nurses to take up ICU training. [Use] Look and learn programmes on hospitals who have ICUs."

"There is a great need for specialist doctors and nurses--such as internal medicine specialist and intensive care nurses."

Comments related to resources such as laboratory support and medications and equipment were also raised:

"Proper and fully equipped laboratory back up [would be useful]. Currently we do not have fully functional biochemistry machines--blood gas [measurement] is essentially non-existent."

"No stable supply of basic hospital drugs/operating theatre equipment and anaesthetic drugs is a major problem."

#### Primary health care

One respondent brought attention to the need to balance a strong focus on preventive and primary care, but to also work towards improving other areas of the hospital:

"In my area of work, most of the causes of death are preventable by good primary health care activities, to which more effort and resources need to be given, while not forgetting improvement of our hospital care facilities."

#### Thinking about critical care

Conducting this research and the survey in itself appeared to have positive outcomes, including inspiring health care workers to think more about critical care in their own health care setting:

"This survey has empowered me to really look at improving this very important unit and the resources needed to care for this category of patient."

## Discussion

The main findings of our survey of 20 health care providers in the Solomon Islands were that critical illness was common, with the four most common conditions leading to critical illness being malaria, diseases of the respiratory system including pneumonia and influenza, diabetes mellitus and tuberculosis. Respondents stated that many basic (e.g. pulse oximeters, oxygen concentrators) and most advanced monitoring, diagnostic, and therapeutic equipment was lacking. Respondents identified the lack of health care personnel as a pressing issue and endorsed the notion that high-quality secondary hospital-based care and primary care should co-exist.

As with all survey research, a limitation of this study is that we collected data on attitudes and beliefs rather than actual clinical practice. There was also some missing data, particularly for questions asking respondents to rank the top five causes of death in patients they treat and rank the top five resources that would most help them treat critically ill patients. Despite steps taken in survey development to maximize clarity, respondents may not have understood the question, or may have found the survey too long. There was also missing data where respondents left questions blank. It was difficult to ascertain if this was because they felt they did not have the appropriate knowledge to answer the question or if it was left blank for another reason.

There is inequity in access to critical care in LMICs throughout the world, yet intensive care medicine is a developing discipline in almost all LMICs [1]. Two major challenges have been identified in providing critical care in LMICs: the first is that there is little infrastructure to deliver healthcare and the second relates to the pre-morbid condition of patients and their disease presentation [13]. More specifically, challenges to the provision of critical care in LMICs include: access to appropriately trained ICU staff, infrastructure including buildings and basic supplies such as water, electricity, oxygen and compressed air, technical services such as medical and nursing equipment, transportation, and supporting disciplines including laboratory, radiology, surgery and transfusion service [1,13]. Challenges also include the limiting factors of intensive care medicine such as poor health status and delay in presentation to medical care [1,13]. We have identified that many of these challenges are also barriers to providing critical care in the Solomon Islands.

The WHO states that every hospital where surgery and anaesthesia are performed should have an ICU, defined as a specialized unit with more skilled nursing care than on general wards, 24 hour monitoring and the provision of oxygen [16]. The Solomon Islands do not have an ICU or any similar facility, and critically ill patients are looked after on general medical wards.

It is thought that hospital mortality can be reduced by simple measures such as increasing nurse:patient ratios, adequate monitoring and greater medical supervision to a percentage of hospital beds [17]. There is a great need for simple, inexpensive therapeutic interventions and methods for monitoring critically ill patients that can be shown to be effective [18]. For example, introducing pulse oximetry together with a good oxygen supply reduced case fatality rates for pneumonia by 35% in Papua New Guinea and the overall mortality risk was significantly reduced by the improved system [19]. An ICU can be created to prioritise basic and inexpensive therapies and fit into a coordinated service that benefits all critically ill patients [17], with the exact personnel and equipment composition of such an ICU depending on local diseases, the hospital's financial and human resources and the community's needs [3].

## Conclusion

There is global recognition that improved critical care could have a significant effect on the burden of disease and effects of ill health. In keeping with current knowledge relating to critical care in LMICs, this survey emphasised the need for basic resources including equipment, trained staff, a proper facility and greater access to medications and blood products. These findings provide a framework upon which to direct future changes in the Solomon Islands, which may emphasize simple measures such as an increased nurse:patient ratio to facilitate closer monitoring of critically ill patients, equipment including pulse oximeters, rational use of oxygen, adequate pain management and blood transfusion. Emphasis should be on the development and acquisition of simple and inexpensive tools rather than complicated equipment, to prevent critical care from unduly diverting resources away from other important parts of the health system.

## Abbreviations

LMICs: Lower and middle income countries; ICU: Intensive care unit; GDP: Gross domestic product; LDC: Least developed country; UNDP: United Nations Development Program; WHO: World health organisation; NA: Not applicable.

## Competing interests

The authors declare that they have no competing interests.

## Authors' contributions

MW participated in design and coordination of the study, collected and analysed the data, and drafted the manuscript. ALCM conceived of the study idea, and participated in its design and coordination and drafted the manuscript. RAF and NKJA designed the survey. All authors contributed to the review and revisions of the manuscript. All authors read and approved the final manuscript.

## Pre-publication history

The pre-publication history for this paper can be accessed here:

http://www.biomedcentral.com/1472-698X/12/1/prepub

## Supplementary Material

Additional file 1 **Details of surveyed hospitals**. Click here for file
