# Supplementary material for: Critical care resources in the Solomon Islands: a cross-sectional survey
Source: BMC Int Health Hum Rights. 2012 Mar 1;12:1. doi: 10.1186/1472-698X-12-1 (PMC3307438; doi:10.1186/1472-698X-12-1)
Supplement: Additional file 1 — Details of surveyed hospitals. [file 1472-698X-12-1-S1.DOC]

**Additional file 1. Details of surveyed hospitals**

The National Referral Hospital in Honiara has 296 beds, 40-50 doctors, approximately 300 nurses, and is the only tertiary referral hospital for the entire Solomon Islands population. It receives 110,000 outpatient visits each year, of which 50 % are received in the Emergency Department. At the time of administration of the survey (October 2010), there were no formal critical care facilities available at National Referral Hospital, and acutely or critically ill patients received care in the medical ward, or if terminally ill, were sent home to be cared for by their families. It was hoped that in the very near future they would be opening an acute care ward at National Referral Hospital (which opened in November 2010). Although its official opening was in November 2010, there were no patients yet admitted to this ward when one of the authors (MW) visited this ward in early December 2010.

Helena Goldie Hospital is a missionary hospital in Munda, in the Western Province of the Solomon Islands. It cares for a population of approximately 10,000-50,000 people, many of whom live in remote areas accessible only by boat. Helena Goldie Hospital has approximately 78 beds, 4 doctors and 35 nurses. Helena Goldie Hospital has the only two ‘special care beds’ in the Solomon Islands, which are separated from the main part of the general medical ward. These beds are used for those patients deemed to be acutely ill, and are closer to the nurses’ station and provide more privacy than beds in the main part of the medical ward

Gizo Hospital is in Gizo, in the Western Province of the Solomon Islands. It cares for a population of approximately 10,000-50,000 people. Gizo Hospital has approximately 60 beds, 4 doctors and 30-50 nurses. Gizo Hospital has no acute care facilities and critically ill patients are cared for on general wards. Due to damage in the 2007 tsunami, Gizo Hospital is being rebuilt, and it is hoped that there will be an acute care ward in the new hospital, although there may not be adequate resources to run this area as an intensive care unit.
